# Expansion of orderly stacked metakaolinite layers and order destruction using a kaolinite-tetraphenylphosphonium chloride intercalation compound†

**DOI:** 10.1039/d1ra03926a

**Published:** 2021-06-30

**Authors:** Shingo Machida, Ken-ichi Katsumata, Atsuo Yasumori

**Affiliations:** Department of Material Science and Technology, Faculty of Advanced Engineering, Tokyo University of Science 6-3-1 Niijuku Katsushika-ku Tokyo 125-8585 Japan shingo.machida@rs.tus.ac.jp

## Abstract

The expansion of metakaolinite layers with stacking order and the order destruction were examined by the heat treatment of a kaolinite-tetraphenylphosphonium chloride intercalation compound (Kaol-TPhPCl) at 540 °C under a nitrogen atmosphere followed by the manual grinding of the product using a mortar and a pestle. Fourier-transform infrared spectroscopy and solid-state ^27^Al nuclear magnetic resonance spectroscopy with magic angle spinning revealed the kaolinite dehydroxylation. Moreover, the absence of kaolinite diffraction lines and the appearance of the 1.85 nm diffraction line in the X-ray diffraction pattern, together with the observation of the hexagonal plate-like morphology in the field-emission scanning electron microscopy, indicated the kaolinite amorphization with the orderly-stacked layers. These results, along with the disappearance of the 1.85 nm diffraction line upon the manual grinding of heat-treated Kaol-TPhPCl, clearly indicated the formation of expanded metakaolinite layers with stacking order and the subsequent order destruction by manual grinding.

## Abstract 2

Metakaolinite layers were expanded *via* the heat treatment of a kaolinite-tetraphenylphosphonium chloride intercalation compound and the obtained metakaolinite stacking order was destroyed upon manual grinding.

## Graphical abstract

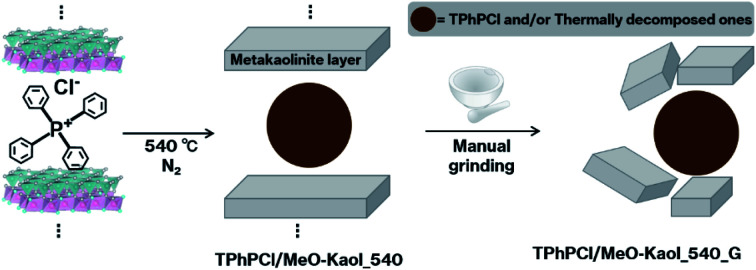

## Introduction

Naturally abundant inorganic solids such as clay minerals, aluminas, and zeolites have been widely investigated for material applications in various fields including environmental and energy sciences.^1–9^ Generally, the adsorption capacities and catalytic activities of these materials are concentrated on the inorganic surfaces,^1–9^ whereas some of them are present inside the particles. In the latter case, to extract the material properties, approaches such as the cleavage of layered inorganic solids^2,3^ can be used, which can be achieved by intercalation and subsequent exfoliation or delamination.^3,10^ Among layered inorganic solids, kaolinite, a layered aluminosilicate clay mineral of formula Al_2_Si_2_O_5_(OH)_4_, comprises stacked neutral layers, each of which consists of an AlO_2_(OH)_4_ octahedral sheet and a SiO_4_ tetrahedral sheet with thickness of 0.72 nm. Additionally, kaolinite can undergo intercalation of organic salts and neutral polar molecules.^11,12^ After intercalation, the exfoliation or delamination of kaolinite layers by mechanochemical grinding proceeds more easily than that of pristine kaolinite,^13,14^ although the AlO_2_(OH)_4_ octahedral surface is essentially inert.^15,16^ Upon the heat treatment of kaolinite 400–750 °C, the heat treated surface, which contains four- or five-coordinated Al centers (Lewis acid sites),^17–19^ undergoes dehydroxylation to form metakaolinite, an amorphous layered aluminosilicate (Al_2_O_3_·2SiO_2_), *i.e.* a dehydrated kaolinite,^17–22^ with multiple structural models^19–22^ whose expansion has never been reported. Not only the expansion but also the destruction of the stacking order of metakaolinite has not been detected on the whole metakaolinite sample. Since the above-mentioned four- or five-coordinated Al centers are generally present on the surfaces of γ-alumina particles^4–6^ whose inner Al cannot be used, inorganic solids with nanometer range thickness bearing four- or five-coordinated Al centers are worth preparing, when it comes to realizing such solids as more reliable solid catalysts and catalyst supports to use their surfaces efficiently.

**Scheme 1:**
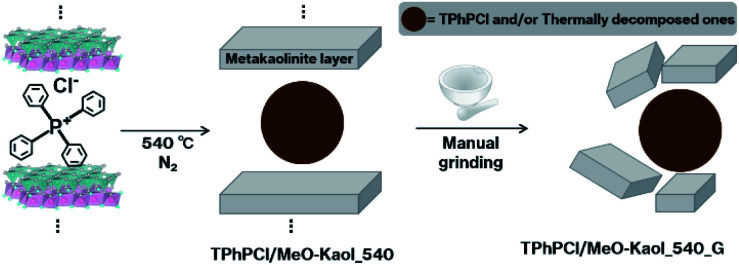
Overview of the present procedures.

Here, we report the expansion of metakaolinite layers with stacking order *via* the heat treatment of a kaolinite-tetraphenylphosphonium chloride (TPhPCl) intercalation compound (Kaol-TPhPCl). The dehydroxylation by heat treatment of kaolinite intercalation compounds generally proceeds at a lower temperature than that of pristine kaolinite, and the intercalated organic molecules are released and/or decomposed, resulting in layer shrinkage.^18^ Organophosphonium salts, especially those bearing phenyl groups,^23^ are relatively stable toward heat treatment.^23–25^ Therefore, the presence of an organophosphonium salt between the layers of kaolinite can be expected to preserve the layer expansion during heat treatment. In the present study, after intercalation of TPhPCl between kaolinite layers using methoxy-modified kaolinite (MeO-Kaol) as an intermediate,^11,26–28^ the product was heated at 540 °C under a nitrogen atmosphere. Since the heated product possessed structure according to the X-ray diffraction (XRD) pattern despite undergoing dehydroxylation and amorphization, this order destruction was examined upon manual grinding (Scheme 1).

## Experimental

### Materials

As a kaolinite sample, a reference clay sample JCSS-1101 obtained from the Clay Science Society of Japan (Kanpaku Mine, Tochigi, Japan) was used. The crystallinity and impurity contents of Kanpaku kaolinite were described elsewhere.^28^ The Hinckley index was 1.0.^28^ Additionally, based on intensities of (020), (110), and (111) reflections of Kanpaku kaolinite,^28^ Aparicio–Galan–Ferrell index (AGFI)^15^ was estimated at 2.2. MeO-Kaol was prepared as previous report.^29^ TPhPCl was obtained from TCI Co., Ltd. Methanol was acquired from Wako Pure Chemical Ind., Ltd. All chemicals were used without further purification.

### Sample preparation

After MeO-Kaol (100 mg) was dispersed in a methanolic solution of TPhPCl (1 mL, 1 mol L^−1^), the dispersion was stirred at room temperature for a day. After the reaction, the resultant solid was centrifuged at 4800 rpm for 1 min, and then dried at 120 °C for 10 min to afford TPhPCl/MeO-Kaol.

Subsequently, TPhPCl/MeO-Kaol (40 mg) was heated to 540 °C using a thermobalance (Shimazu DTG-60) at a heating rate of 10 °C min^−1^ under a nitrogen atmosphere, producing TPhPCl/MeO-Kaol_540.

Then, TPhPCl/MeO-Kaol_540 (20 mg) was manually ground using a mortar and a pestle as follows; when the contact with the pestle did not produce any more changes in the ground powders, the powders were collected from the mortar center by a spatula. The grinding process was conducted four times, affording TPhPCl/MeO-Kaol_540_G. All grinding procedures took approximately 15 min.

### Characterization

The XRD patterns were recorded on a Shimazu XRD-6100 diffractometer operated at 30 mA and 40 kV with monochromatic Cu Kα radiation. Field-emission scanning electron microscopy (FE-SEM) images were recorded on a Zeiss spra40 microscope. Prior to the measurement, the samples were coated with platinum by sputtering. Fourier-transform infrared (IR) spectra (KBr disk method) were recorded on a JASCO FT/IR-4100 spectrometer with a resolution of 2.0 cm^−1^. Solid-state ^27^Al nuclear magnetic resonance (NMR) spectra were recorded on a Bruker ADVANCE NEO 400 spectrometer at 103.15 MHz, and were obtained using magic angle spinning (MAS) techniques with a pulse delay of 1 s and a spinning rate of 8 kHz. Solid-state ^29^Si NMR spectra were recorded on a JEOL ECX-400 spectrometer at 79.42 MHz, and were obtained using MAS technique with a pulse delay of 5 s and cross-polarization (CP) at a contact time of 5 ms.

## Results

**Fig. 1:**
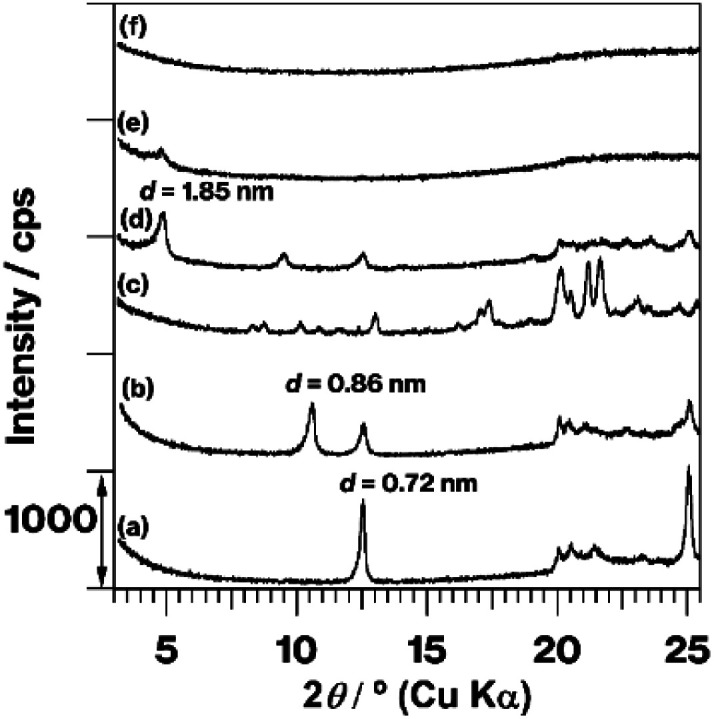
XRD patterns of (a) kaolinite, (b) MeO-Kaol, (c) TPhPCl, (d) TPhPCl/MeO-Kaol, (e) TPhPCl/MeO-Kaol_540, and (f) TPhPCl/MeO-Kaol _540_G.

Fig. 1 shows the XRD patterns of kaolinite, MeO-Kaol, TPhPCl, TPhPCl/MeO-Kaol, TPhPCl/MeO-Kaol_540, and TPhPCl/MeO-Kaol_540_G. The XRD pattern of TPhPCl/MeO-Kaol (Fig. 1d) shows the diffraction line with a *d* value of 1.85 nm along with the disappearance of the diffraction line with a *d* value of 0.86 nm due to MeO-Kaol (Fig. 1b), indicating an increase in the basal spacing from 0.86 to 1.85 nm. No diffraction line is observed in the 2*θ* angle range of 3–8° in the XRD pattern of TPhPCl (Fig. 1c). The diffraction line with a *d* value of 0.72 nm due to pristine kaolinite (Fig. 1a) is also observed in the XRD patterns of MeO-Kaol and TPhPCl/MeO-Kaol; this is in agreement with the well-known incomplete intercalation of guest species between the layers of kaolinite.^30^ The XRD pattern of TPhPCl/MeO-Kaol_540 (Fig. 1e) also exhibits the diffraction line with a *d* value of 1.85 nm along with the disappearance of the diffraction lines due to TPhPCl/MeO-Kaol in the 18–26 two *θ* angle range. The 1.85 nm diffraction line clearly disappears in the XRD pattern of TPhPCl/MeO-Kaol_540_G (Fig. 1f).

Fig. S1† shows the FE-SEM images of kaolinite, MeO-Kaol, TPhPCl/MeO-Kaol, TPhPCl/MeO-Kaol_540, and TPhPCl/MeO-Kaol_540_G. As can be seen in Fig. S1a,† kaolinite exhibits a hexagonal plate-like morphology,^25,28^ which is also observed in the images of MeO-Kaol, TPhPCl/MeO-Kaol, TPhPCl/MeO-Kaol_540, and TPhPCl/MeO-Kaol_540_G (Fig. S1b–e†). Among them, the image of TPhPCl/MeO-Kaol_540_G (Fig. S1e†) displays smaller particles than those observed in the image of TPhPCl/MeO-Kaol_540 (Fig. S1d†). It should be noted that metakaolinite also showed a hexagonal plate-like morphology.^20,21^

**Fig. 2:**
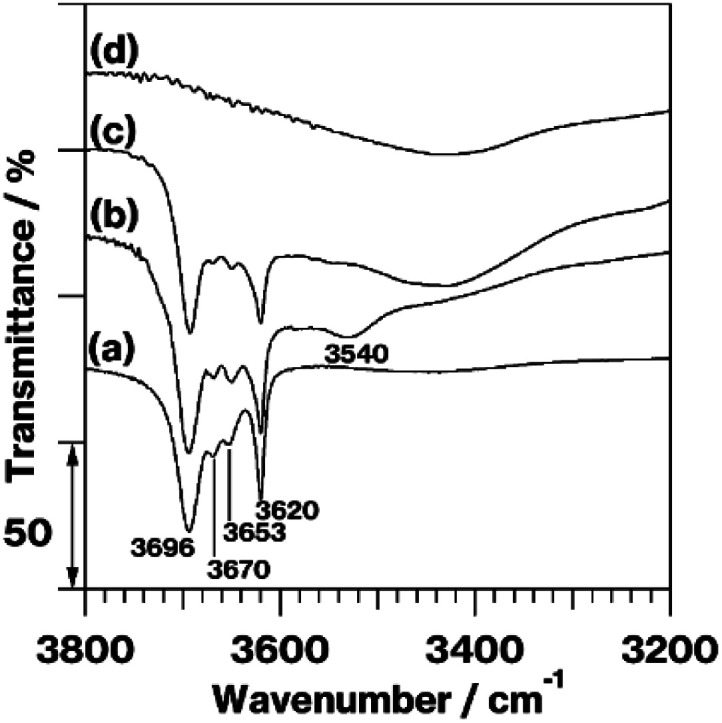
IR spectra in the OH stretching region of (a) kaolinite, (b) MeO-Kaol, (c) TPhPCl/MeO-Kaol, and (d) TPhPCl/MeO-Kaol_540.

Fig. 2 shows the IR spectra in the OH stretching region of kaolinite, MeO-Kaol, TPhPCl/MeO-Kaol, and TPhPCl/MeO-Kaol_540. The spectrum of kaolinite (Fig. 2a) exhibits four OH stretching bands, among which the 3620 cm^−1^ band is assignable to inner-layer hydroxyl groups, and the 3696, 3670, and 3653 cm^−1^ bands can be attributed to inter-layer hydroxyl groups.^11,31,32^ The former band is not perturbed, whereas the latter bands are affected by intercalation of guest species; compared with the spectrum of kaolinite, the relative intensities of 3696, 3670, and 3653 cm^−1^ bands decrease with respect to that of 3620 cm^−1^ band.^11,31,32^ Compared with the spectrum of kaolinite (Fig. 2a), the spectrum of MeO-Kaol (Fig. 2b) displays a decrease in the relative intensities of 3696, 3670, and 3653 cm^−1^ bands with respect to that of the 3620 cm^−1^ band along with the appearance of 3540 cm^−1^ band which is ascribable to the interactions of inter-layer hydroxyl groups of kaolinite with the intercalated water molecules.^29^ The latter band disappears in the spectrum of TPhPCl/MeO-Kaol (Fig. 2c) which shows the decrease in the relative intensities of 3696, 3670, and 3653 cm^−1^ bands with respect to that of 3620 cm^−1^ band as compared with the spectra of kaolinite and MeO-Kaol (Fig. 2a and b). The IR profile of TPhPCl/MeO-Kaol is similar to those of kaolinite-organophosphonium salt intercalation compounds.^25^ The four OH stretching bands observed in the spectrum of TPhPCl/MeO-Kaol (Fig. 2c) disappear in the spectrum of TPhPCl/MeO-Kaol_540 (Fig. 2d).

Fig. S2† shows the IR spectra of kaolinite, MeO-Kaol, TPhPCl/MeO-Kaol, TPhPCl and, TPhPCl/MeO-Kaol_540 in the 1600–800 cm^−1^ range. The spectrum of TPhPCl/MeO-Kaol (Fig. S2c†) exhibits C–C stretching bands^33^ at 1587, 1485, 1340, and 1313 cm^−1^ and a *P*-phenyl stretching band^34^ at 1439 cm^−1^ whose positions are the same as those observed in the spectrum of TPhPCl (Fig. S2d†). Compared with the latter spectrum, the *P*-phenyl stretching band^34^ at 1439 cm^−1^ is also observed in the spectrum of TPhPCl/MeO-Kaol_540 (Fig. S2e†), whereas the C–C stretching bands^33^ at 1587, 1485, 1340, and 1313 cm^−1^ disappear.

**Fig. 3:**
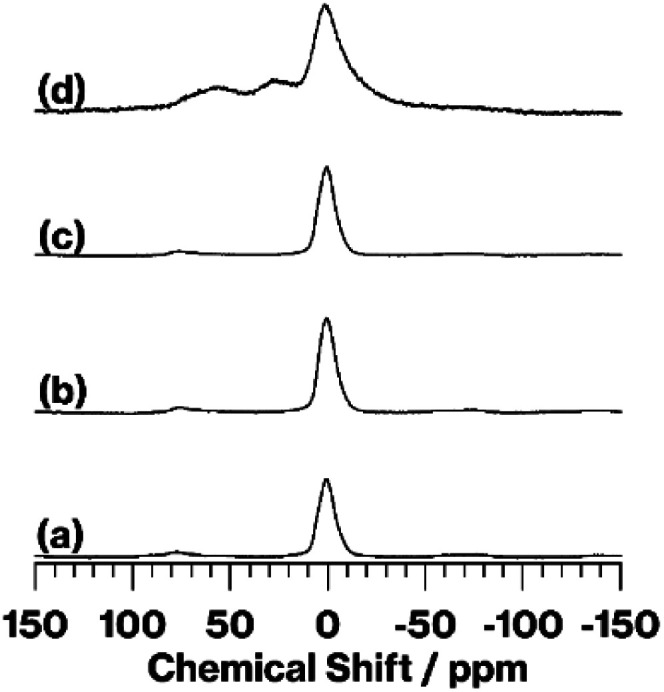
^27^Al MAS NMR spectra of (a) kaolinite, (b) MeO-Kaol, (c) TPhPCl/MeO-Kaol, and (d) TPhPCl/MeO-Kaol_540.

Fig. 3 shows the ^27^Al MAS NMR spectra of kaolinite, MeO-Kaol, TPhPCl/MeO-Kaol, and TPhPCl/MeO-Kaol_540. The spectrum of kaolinite (Fig. 3a) exhibits a broad signal at 0 ppm, which is assignable to six-coordinated Al in the AlO_2_(OH)_4_ sheets.^17,28^ Similar signals are observed in the spectra of MeO-Kaol and TPhPCl/MeO-Kaol (Fig. 3b and c). Compared with the spectrum of TPhPCl/MeO-Kaol (Fig. 3c), the spectrum of TPhPCl/MeO-Kaol (Fig. 3d) displays the same broad signal at 0 ppm along with broad signals at 55 and 30 ppm, which can be attributed to four- and five-coordinated Al,^16,17^ respectively.

Fig. S3† shows ^29^Si CP/MAS NMR spectra of kaolinite, MeO-Kaol, TPhPCl/MeO-Kaol, and TPhPCl/MeO-Kaol_540. The spectra of kaolinite, MeO-Kaol and TPhPCl/MeO-Kaol are observed in the −91 to −92 ppm range (Fig. S3a–c†), while that of TPhPCl/MeO-Kaol_540 displays the relatively broad signal at around −94 ppm (Fig. S3d†).

## Discussion

The XRD patterns (Fig. 1b–d), IR spectra (Fig. 2a–c and S2a–c†), and FE-SEM images (Fig. S1a–c†) of the product reveal that TPhPCl molecules are intercalated between kaolinite layers forming a kaolinite-TPhPCl intercalation compound (Kaol-TPhPCl). It is well-known that liquid guests and solutions with high guest concentrations are required for the kaolinite intercalation reaction to proceed.^11,12^ Generally, the obtained intercalation compounds accompany with neat liquids and bulk solids, since the guest species are easily deintercalated upon washing of the kaolinite intercalation compounds. Therefore, the characteristics of neat liquids and bulk solids at the outer surfaces of kaolinite intercalation compounds are unavoidable. It should be noted that tetraphenylphosphonium bromide (TPhPBr), a similar guest specie to TPhPCl, was intercalated between the layers of kaolinite using a kaolinite-tetrabutylphosphonium bromide (TBPBr) intercalation compound (Kaol-TBPBr) as an intermediate which has showed successful organic salt exchange reactions.^25,35,36^ Kaol-TBPBr featured the presence of a part of rolled-up kaolinite layers when Kaol-TBPBr was prepared using a kaolinite-dimethylsulfoxide intercalation compound as an intermediate. The use of MeO-Kaol as an intermediate for intercalation of TPhPCl thus succeeds in the preventing kaolinite layer rolling up.

The XRD patterns (Fig. 1) reveal the occurrence of kaolinite amorphization. Additionally, hexagonal plate-like morphologies are preseved based on the FE-SEM images (Fig. S2a–d†). The IR spectra in the OH stretching region (Fig. 2) and the ^27^Al MAS NMR spectra (Fig. 3) indicate that kaolinite dehydroxylation occurs. The spectrum broadening along with a slight upfield shift due to a part of structural conversion of Si environment from Q^3^ to Q^4^ in SiO_4_ sheets, which was discussed in the previous report,^18^ is clearly shown in the ^29^Si CP/MAS NMR spectra (Fig. S3c and d†). These results clearly reveal the conversion of the kaolinite intercalation compound into metakaolinite by TPhPCl/MeO-Kaol heat treatment at 540 °C under a nitrogen atmosphere. The heat-treated product, TPhPCl/MeO-Kaol_540, contains *P*-phenyl groups (Fig. S2e†), which might be ascribable to the presence of TPhPCl and/or thermally-decomposed products as following reasons; (1) the organic compounds were gradually decomposed by increasing the thermal treatment temperature *via* the formation of carbonaceous materials;^17^ (2) the thermally-decomposed tetraphenylphosphonium cations contained *P*-phenyl groups.^23^ The reactions between kaolinite layers and TPhPCl and its thermally-decomposed products seem to hardly occur, since there are no functional groups such as OH and OR (R represents organic groups) groups^27,28^ in the thermally-decomposed products.^17^ Despite the above-mentioned conversion, the XRD pattern of TPhPCl/MeO-Kaol_540, the present metakaolinite, shows the 1.85 nm diffraction line (Fig. 1e), indicating the presence of an ordered structure to a certain extent. It is well-known that metakaolinite possesses a layered structure^18,20–22^ with a lateral atom arrangement of kaolinite layers with approximately 0.44 nm equidistance.^21^ The 1.85 nm repeating distance, therefore, reveals the presence of layered periodicity in the present metakaolinite.

Regarding the structure of the metakaolinite layer, multiple structural models have been proposed in which the thickness range from 0.62 to 0.74 nm (ref. 19 and 22) depending on the dehydroxylation degree, which can be continuously changed by temperature. Therefore, the structure of the metakaolinite layers cannot be explained by a single model.^22^ Meanwhile, transmission electron microscopy (TEM) observation of metakaolinite showed the presence of a few interlayers with approximately 1.2 nm periodicity because of polymerization of two kaolinite layers;^21^ the thickness of one metakaolinite layer thickness was thus approximately 0.60 nm. These studies focused on metakaolinite obtained from pristine kaolinite whose dehydroxylation starts near defects such as dislocations, stacking faults, and edges.^21^ Among them, the stacking fault of pristine kaolinite seems to be different from those of kaolinite intercalation compounds in terms of the expansion degree of the kaolinite, which is dominated by the guest species and their thermal stabilities. Therefore, the dehydroxylation degree and process of kaolinite intercalation compounds are thus different from those of pristine kaolinite. The metakaolinite layers are ambiguous, while the present 1.85 nm repeating distance is larger than the proposed metakaolinite layer thicknesses of 0.60–0.74 nm, some of which remained a portion of OH groups in pristine kaolinite.^22^ Additionally, metakaolinite remained a portion of kaolinite lateral atom arrangement in a relatively shorter degree as described before (see the previous paragraph).^21^ The same repeating distance value of 1.85 nm between TPhPCl/MeO-Kaol and TPhPCl/MeO-Kaol_540 is therefore one of possible results. Consequently, it seems reasonable to conclude that the 1.85 nm value observed in the present study corresponds to the expanded metakaolinite layers. Meanwhile, the weak interaction of kaolinite with TPhPCl may thus likely to be remained in TPhPCl/MeO-Kaol_540. The minute amounts of hydroxyls in metakaolinite were generally detected using differential thermal analysis at around 980 °C.^37^ Such the temperature shows the risk to shrinkage of expanded metakaolinite layer due to complete decomposition of TPhPCl. The detect of OH groups, which are weakly interacted with TPhPCl, would require deuterated chemicals and kaolinite;^38,39^ thus, this could be another study.

Since the 1.85 nm diffraction line can be attributed to the expanded metakaolinite layers, the XRD patterns (Fig. 1e and f) and FE-SEM images (Fig. S1d and e†) reveal the destruction of the stacking order accompanied by particle breaking. Even though metakaolinite particles can be broken by manual grinding of metakaolinite obtained from pristine kaolinite, the stacking order destruction on the whole sample cannot be detected because no diffraction line is observed in the XRD pattern of the original metakaolinite. Although metakaolinite can be obtained after grinding of kaolinite and/or its intercalation compounds and subsequent heat treatment, the grinding for approximately 15 min is more facile; no disappearance of the diffraction line of pristine kaolinite was observed upon mechanochemical grinding using a planetary mill for 6 h.^13,14^ Additionally, diffraction lines due to expanded kaolinite layers in the intercalation compounds appeared upon the milling for 2–6 h.^13,14^

The intercalated carbonaceous material derived from acrylamide thermal polymerization at 620 °C partially suppressed the conversion of kaolinite into metakaolinite, because of the hydrogen bond formation between the C=O groups of the carbonaceous material and the interlayer hydroxy groups of kaolinite.^17^ By contrast, the weak interaction of TPhPCl and the interlayer hydroxyls of kaolinite, which is similar to those of kaolinite-organic salt intercalation compounds,^25,27,40,41^ is thus likely to facilitate the conversion of kaolinite into metakaolinite, as can be concluded from the following results; (1) the XRD pattern of the present metakaolinite shows no diffraction lines attributed to kaolinite (Fig. 1e), whereas pristine kaolinite heated at 540 °C showed kaolinite diffraction lines;^17^ (2) the ^27^Al MAS NMR spectrum of the present metakaolinite (Fig. 3d) exhibits larger four- and five-coordinated Al signals than those of pristine kaolinite heated at 540 °C.^17^ The difference in degree of amorphization between an expanded and non-expanded metakaolinite is therefore worthy to be further investigated using techniques of localized characteristics such as TEM analysis,^21^ and we will do our best to continue our study.

## Conclusions

We have clearly demonstrated the expansion of metakaolinite layers with stacking order and the order destruction. Expanded metakaolinite layers with 1.85 nm periodicity were successfully obtained by the heat treatment of Kaol-TPhPCl at 540 °C under a nitrogen atmosphere. The stacking order destruction along with particle breaking easily proceeded upon manual grinding of the expanded metakaolinite using a mortar and a pestle. The present study thus displays characteristics on the whole metakaolinite intercalation compound sample. Additionally, the present study could provide a method for the effective use of the metakaolinite surfaces.

## Author contributions

Shingo Machida: conceptualization, data curation, investigation, project administration, writing-original draft, supervision. Ken-ichi Katsumata: project administration. Atsuo Yasumori: project administration.

## Conflicts of interest

There are no conflicts to declare.

## Supplementary Material

RA-011-D1RA03926A-s001
