# Supplementary material for: Expansion of orderly stacked metakaolinite layers and order destruction using a kaolinite-tetraphenylphosphonium chloride intercalation compound
Source: RSC Adv. 2021 Jun 30;11(37):23090–4. doi: 10.1039/d1ra03926a (PMC9034375; doi:10.1039/d1ra03926a)
Supplement: RA-011-D1RA03926A-s001 [file RA-011-D1RA03926A-s001.pdf]

Electronic Supplementary Material (ESI) for RSC Advances.
This journal is © The Royal Society of Chemistry 2021

Electrical Supporting Information

Expansion of orderly stacked metakaolinite layers and order destruction using a kaolinite-tetraphenylphosphonium chloride intercalation compound

Shingo Machida[a], Ken-ichi Katsumata[a], Atsuo Yasumori[a]*

[a]Department of Material Science and Technology, Faculty of Advanced Engineering, Tokyo University of Science,

6-3-1 Niijuku, Katsushika-ku, Tokyo 125-8585, Japan

*E-mail: shingo.machida@rs.tus.ac.jp

Figures

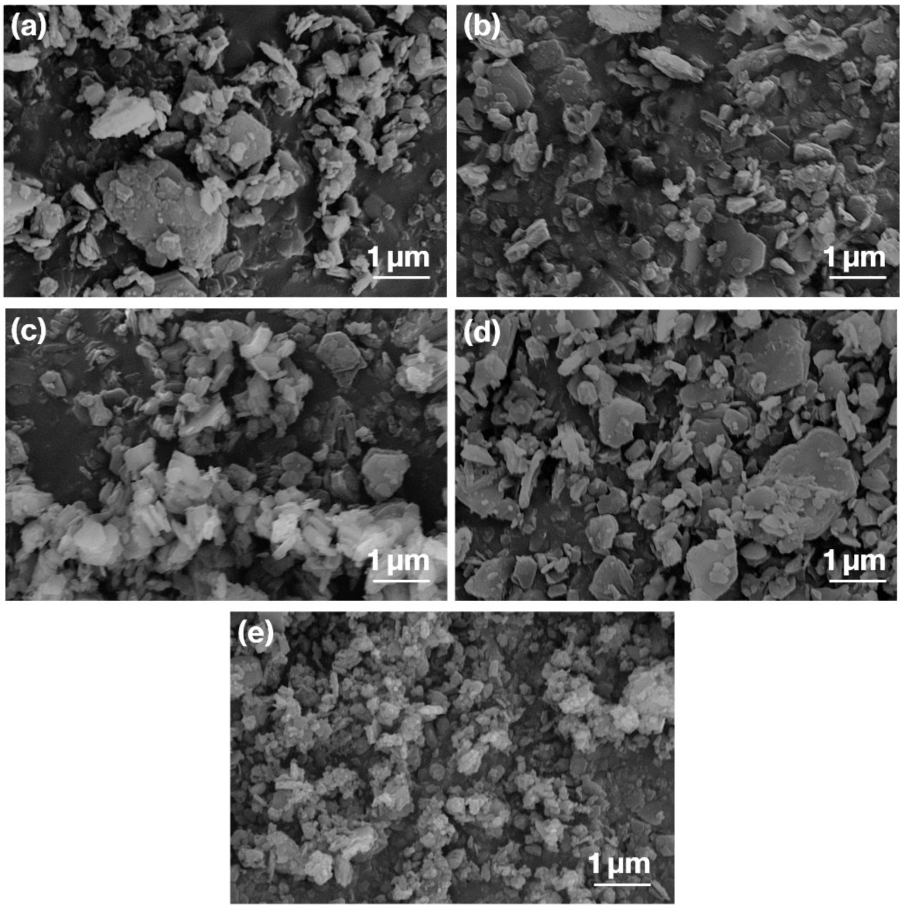

Figure S1. FE-SEM images of (a) kaolinite, (b) MeO-Kaol, (c) TPhPCl/MeO-Kaol, (d) TPhPCl/MeO-Kaol_540 and (e) TPhPCl/MeO-Kaol _540_G.

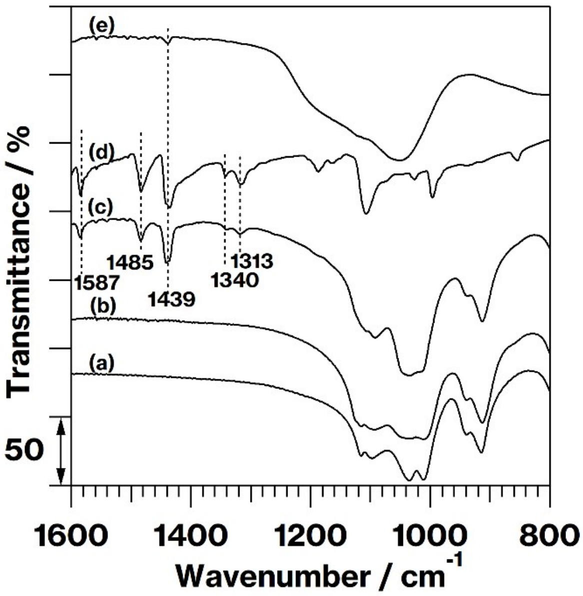

Figure S2. IR spectra in the 1600-400 cm^{-1} range of (a) kaolinite, (b) MeO-Kaol, (c) TPhPCl/MeO-Kaol, (d) TPhPCl and (e) TPhPCl/MeO-Kaol_540.

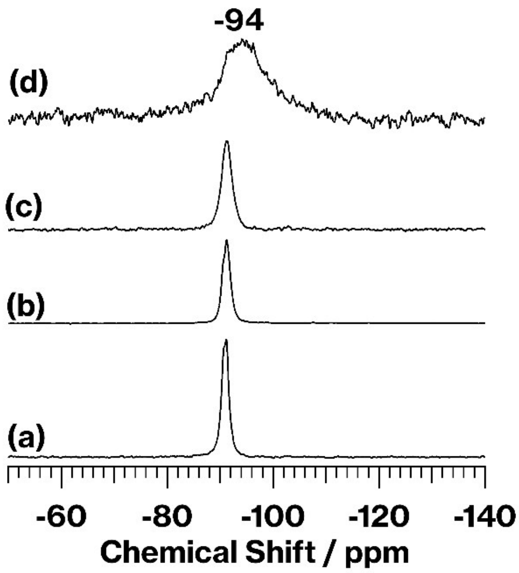

Figure S3. ^{29}Si CP/MAS NMR spectra of (a) kaolinite, (b) MeO-Kaol, (c) TPhPCl/MeO-Kaol and (d) TPhPCl/MeO-Kaol_540.
